# Supplementary material for: Characterization of an eutherian gene cluster generated after transposon domestication identifies Bex3 as relevant for advanced neurological functions
Source: Genome Biol. 2020 Oct 26;21:267. doi: 10.1186/s13059-020-02172-3 (PMC7586669; doi:10.1186/s13059-020-02172-3)
Supplement: Supplementary file 1 — Additional file 1: Fig. S1 The Tceal7 gene is derived from the domestication of L1 retrotransposon fragments. Fig. S2 The Bex/Tceal gene cluster was established before the diversification of extant eutherians. Fig. S3 Highly diverged BEX/TCEAL proteins share a coiled coil domain. Fig. S4 BEX/TCEAL proteins might have inherited some of their structural properties from the ancestral transposon. Fig. S5 Selection pressure analyses reveal signatures of positive selection in the Bex/Tceal genes. Fig. S6 A BGW-like sequence was already present in the GLA promoter of the last therian common ancestor. Fig. S7 Bex/Tceal genes show tissue-enriched expression patterns during development. Fig. S8 Bex3 and Tceal7 genes, but not the ancestral HALEX element, induce cell proliferation in chicken neural tube. Fig. S9 The deletions introduced using CRISPR-Cas9 technology can be observed in the mRNA expressed from the Bex3 mutant alleles. Fig. S10 CRISPR-Cas9-generated Bex3 mutant alleles show subtle skull abnormalities. Fig. S11 Bex3 mutant mice show normal acoustic startle reflex. Fig. S12 Bex3 deficiency leads to aberrant mTOR signaling in the brain. Table S1 Coding genes putatively derived from transposable elements in the human and mouse genomes. Table S2 Altered expression of BEX and TCEAL genes in subjects with autism spectrum disorder or schizophrenia. Table S3 Enrichment of differential gene expression in BEX and TCEAL gene families in subjects with autism spectrum disorder or schizophrenia. Table S4 Primers and reconstructed gene sequences used in this work. Supplementary references. [file 13059_2020_2172_MOESM1_ESM.pdf]

Characterization of an eutherian gene cluster generated after transposon domestication identifies *Bex3* as relevant for advanced neurological functions

Enrique Navas-Pérez†, Cristina Vicente-García†, Serena Mirra†, Demian Burguera, Noèlia Fernàndez-Castillo, José Luis Ferrán, Macarena López-Mayorga, Marta Alaiz-Noya, Irene Suárez-Pereira, Ester Antón-Galindo, Fausto Ulloa, Carlos Herrera-Úbeda, Pol Cuscó, Rafael Falcón-Moya, Antonio Rodríguez-Moreno, Salvatore D'Aniello, Bru Cormand, Gemma Marfany, Eduardo Soriano, Ángel M. Carrión, Jaime J. Carvajal*, Jordi Garcia-Fernàndez*

† These authors contributed equally to this work.

* Corresponding authors. E-mails: jcarvajal@csic.es (J.J.C), jordigarcia@ub.edu (J.G.-F.).

Supplementary information

Table of contents:

Fig. S1 The *Tceal7* gene is derived from the domestication of L1 retrotransposon fragments.

Fig. S2 The *Bex/Tceal* gene cluster was established before the diversification of extant eutherians.

Fig. S3 Highly diverged BEX/TCEAL proteins share a coiled-coil domain.

Fig. S4 BEX/TCEAL proteins might have inherited some of their structural properties from the ancestral transposon.

Fig. S5 Selection pressure analyses reveal signatures of positive selection in the *Bex*/*Tceal* genes.

Fig. S6 A BGW-like sequence was already present in the *GLA* promoter of the last therian common ancestor.

Fig. S7 *Bex*/*Tceal* genes show tissue-enriched expression patterns during development.

Fig. S8 *Bex3* and *Tceal7* genes, but not the ancestral HALEX element, induce cell proliferation in chicken neural tube.

Fig. S9 The deletions introduced using CRISPR-Cas9 technology can be observed in the mRNA expressed from the *Bex3* mutant alleles.

Fig. S10 CRISPR-Cas9-generated *Bex3* mutant alleles show subtle skull abnormalities.

Fig. S11 *Bex3* mutant mice show normal acoustic startle reflex.

Fig. S12 *Bex3* deficiency leads to aberrant mTOR signalling in the brain.

Table S1 Coding genes putatively derived from transposable elements in the human and mouse genomes.

Table S2 Altered expression of *BEX* and *TCEAL* genes in subjects with autism spectrum disorder or schizophrenia.

Table S3 Enrichment of differential gene expression in *BEX* and *TCEAL* gene families in subjects with autism spectrum disorder or schizophrenia.

Table S4 Primers and reconstructed gene sequences used in this work.

Supplementary references

Fig. S1 The *Tceal7* gene is derived from the domestication of L1 retrotransposon fragments. Maximum-likelihood phylogenetic trees constructed using the nucleotidic sequences of ancient eutherian and metatherian L1 subfamilies, and the correspoding highlighted fragment of the *Tceal7* gene of three eutherian species: Hsa, *Homo sapiens*; Eca, *Equus caballus*; Laf, *Loxodonta africana*. The trees were inferred using IQ-TREE. The statistical support values are SH-aLRT and ultrafast bootstrap (UFBoot). The scale bar represents the expected number of nucleotide substitutions per site.

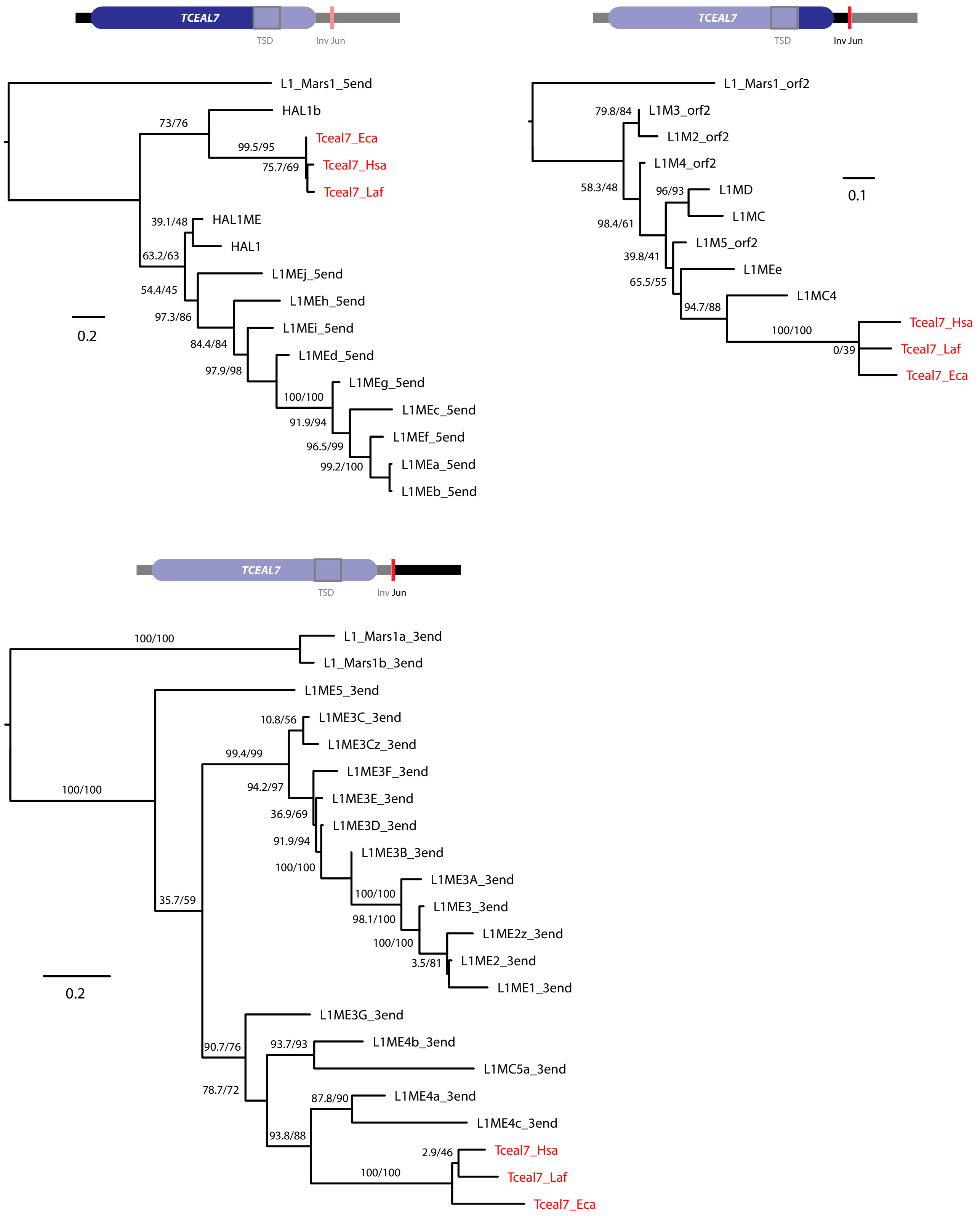

Fig. S2 The *Bex/Tceal* gene cluster was established before the diversification of extant eutherians. Genes are represented with triangles and pseudogenes with empty triangles. The transparent pseudogenes in armadillo's row were found in the genome of another xenarthran: Hoffmann's two-toed sloth (*Choloepus hoffmanni*). The phylogenetic tree of eutherian species was constructed according to previous studies [1].

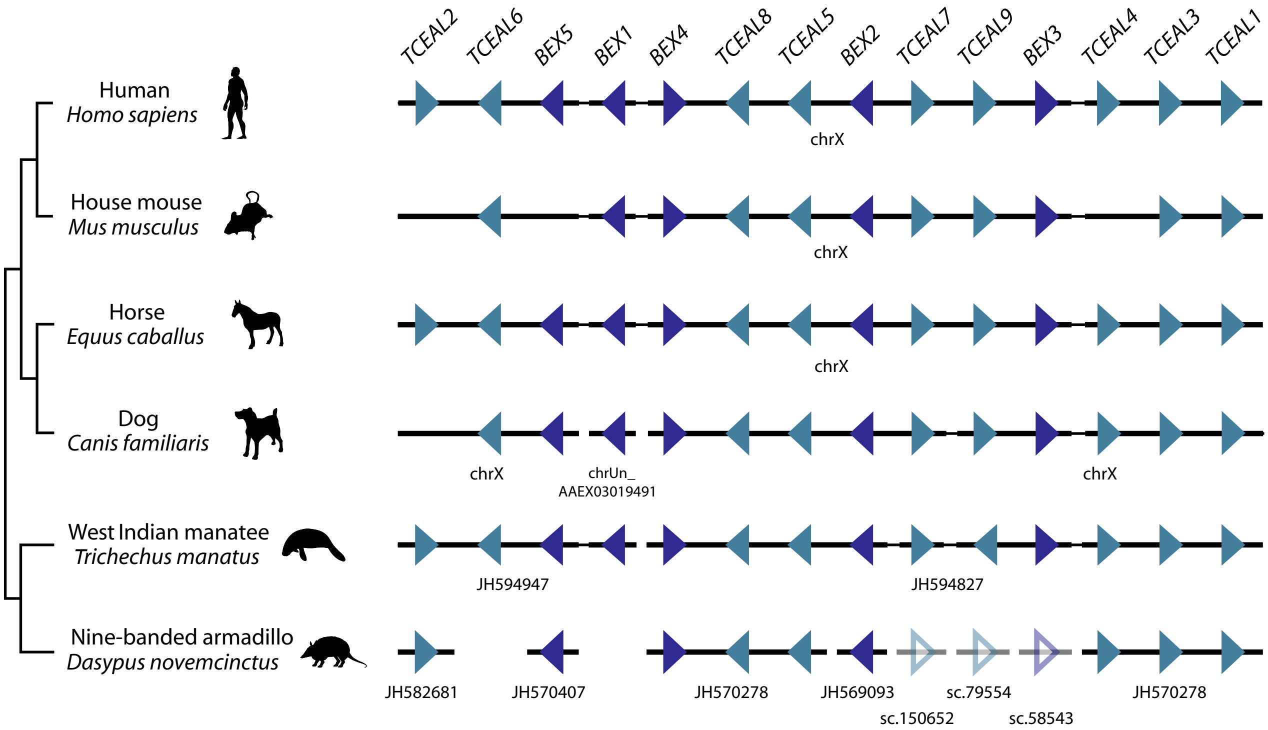

Fig. S3 Highly diverged BEX/TCEAL proteins share a coiled-coil domain. **A** Protein alignment of human BEX and TCEAL proteins. Shaded in soft red and strong red are regions predicted by PCOILS to form coiled coils with a probability of 0.5-0.9 and > 0.9, respectively. **B** Maximum-likelihood phylogenetic tree constructed using the sequences of BEX and TCEAL proteins from eight eutherian species: dog, mouse, human, horse, cow, African bush elephant, nine-banded armadillo and West Indian manatee. The tree was inferred using IQ-TREE. The statistical support values are SH-aLRT and ultrafast bootstrap (UFBoot). The scale bar represents the expected number of substitutions per site.

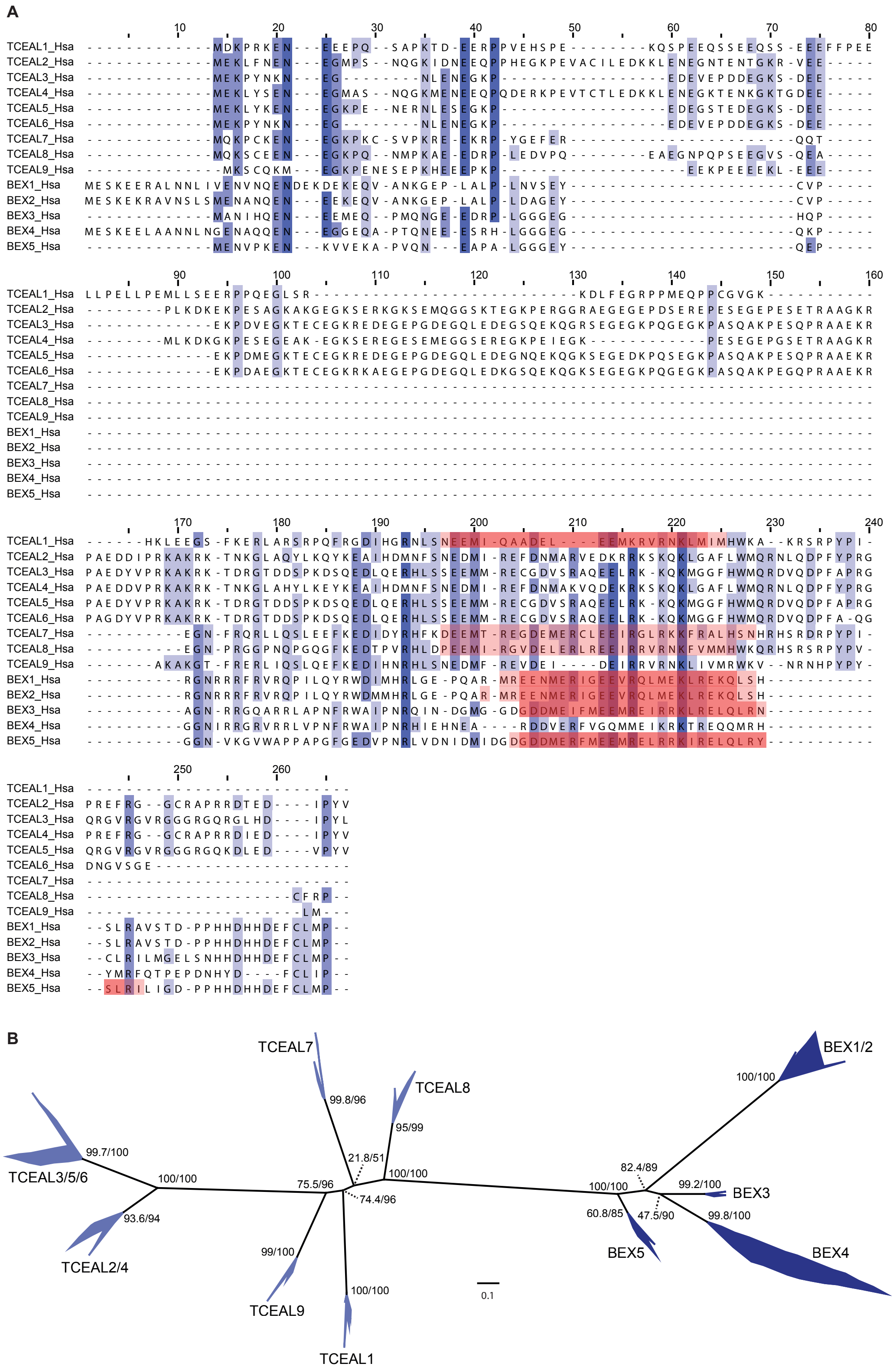

Fig. S4 BEX/TCEAL proteins might have inherited some of their structural properties from the ancestral transposon. Protein disorder and α-helix predictions for the N-terminal end of HAL1b and for human BEX and TCEAL proteins.

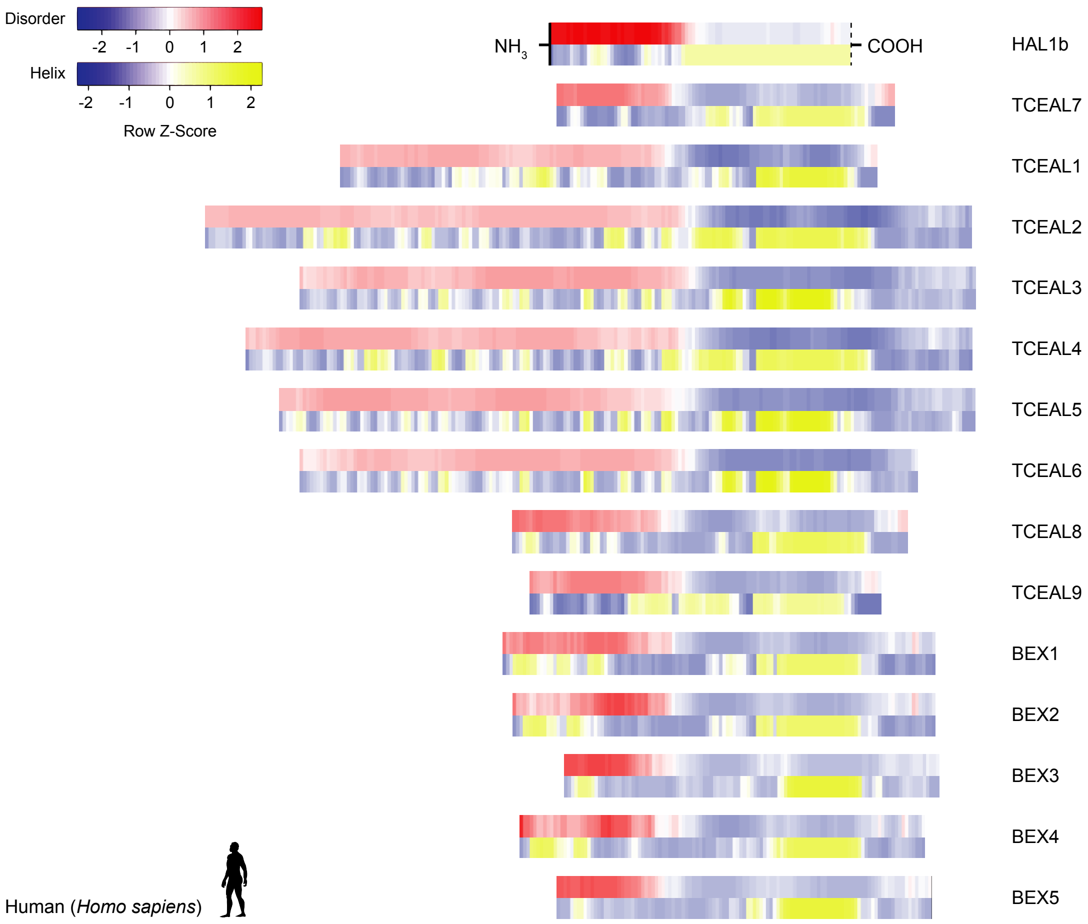

Fig. S5 Selection pressure analyses reveal signatures of positive selection in the *Bex/Tceal* genes. MEME and aBSREL methods were used to infer selection pressures acting on **A** the *Bex* genes using *Tceal7* as an outgroup, and **B** the *Tceal* genes using *Bex5* as an outgroup. *Bex/Tceal* genes that homogenize their coding regions through gene conversion were excluded from the analysis. Amino acid sequences from eight eutherian species (Hsa, *Homo sapiens*; Eca, *Equus caballus*; Bta, *Bos taurus*; Laf, *Loxodonta africana*; Tma, *Trichechus manatus*; Cfa, *Canis familiaris*; Dno, *Dasypus novemcinctus*; Mmu, *Mus musculus*) were used to build the alignments and the phylogenetic trees. Branches under positive selection according to aBSREL ($P < 0.05$) are shown in red on the phylogenetic trees, and the ω1 and ω2 values indicate that there are two ω classes in a given branch with different selective pressures. Sites in the alignment under positive selection according to MEME ($P < 0.05$) are shown highlighted in grey on the right. Phylogenetic trees were inferred using IQ-TREE. The statistical support values are SH-aLRT and ultrafast bootstrap (UFBoot). The scale bar represents the expected number of substitutions per site.

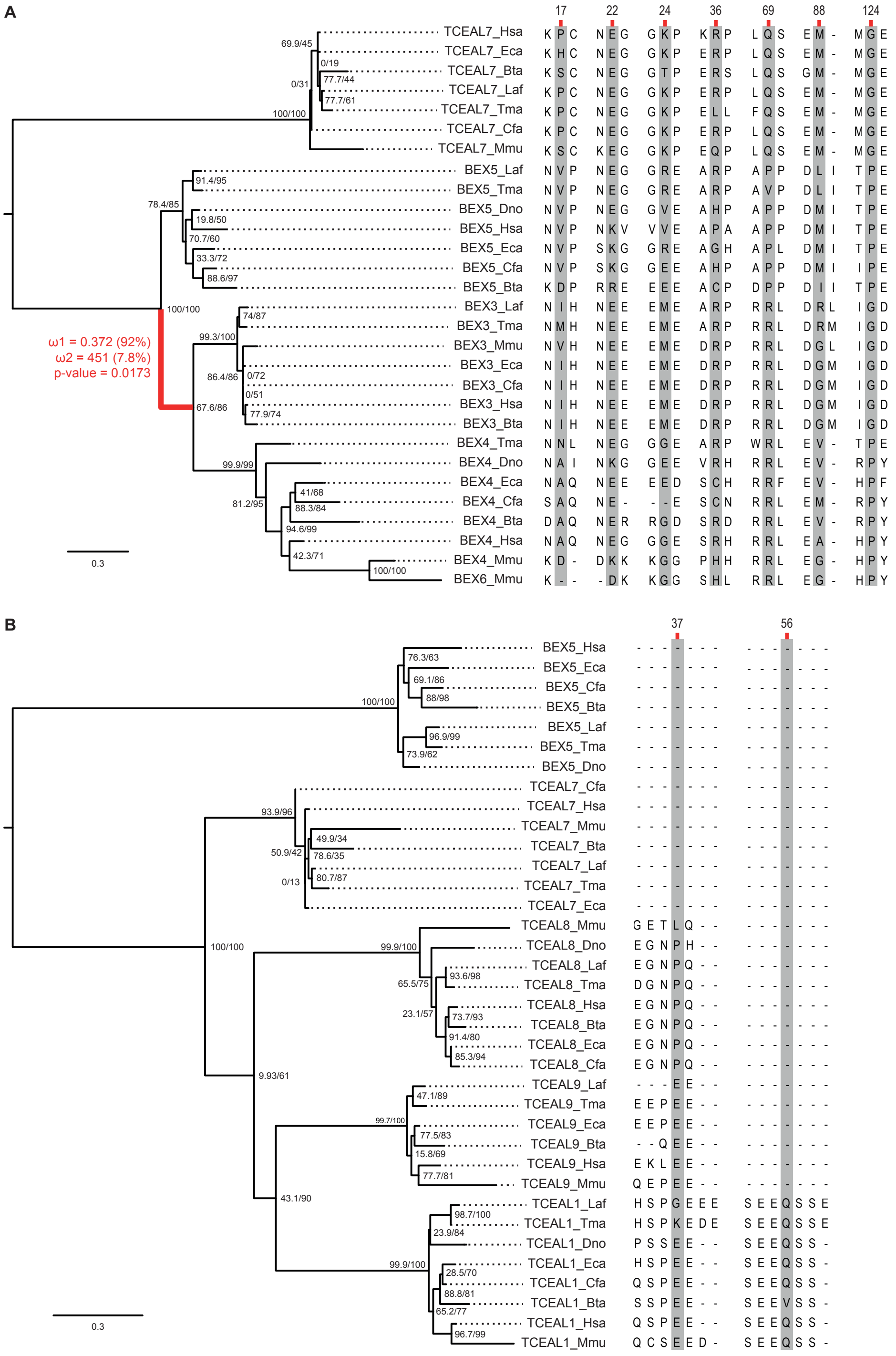

Fig. S6 A BGW-like sequence was already present in the *GLA* promoter of the last therian common ancestor. Alignment of the *Hnrnph2* BGW motif and its orthologous region in marsupial species. Shaded in red, the conserved E-box sequence.

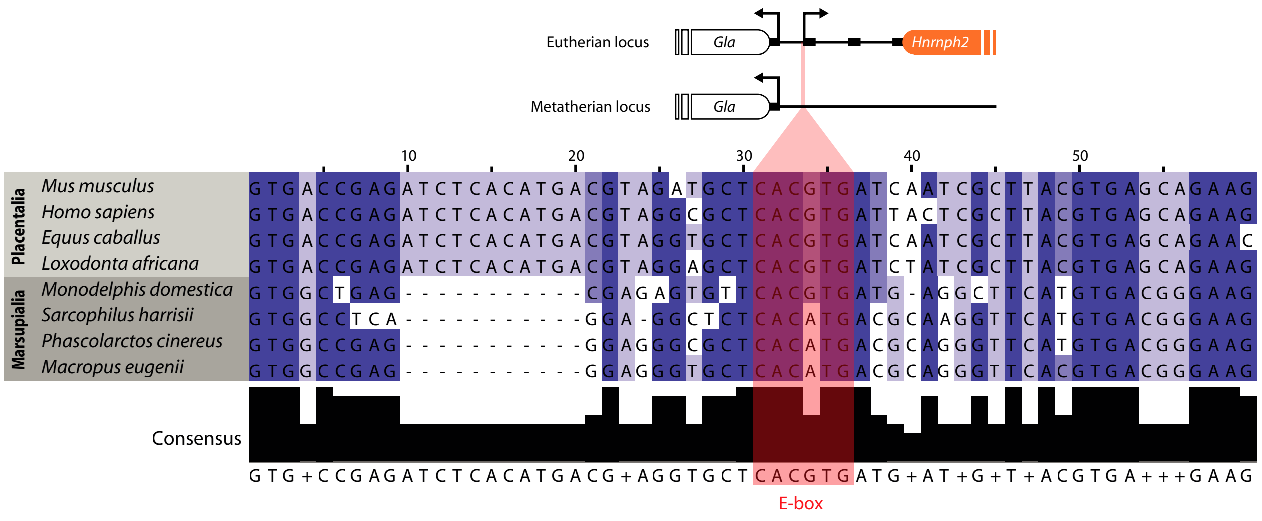

Fig. S7 *Bex/Tceal* genes show tissue-enriched expression patterns during development. Expression patterns of *Bex/Tceal* genes using *in situ* hybridization (ISH) in E13.5 mouse embryos. Whole sagittal sections and details showing expression in specific tissues are shown. ca, cartilage; drg, dorsal root ganglion; ge, gut epithelium; gg gasserian ganglion; he, heart; ki, kidney; le, lens; li, liver; lu, lung; oe, olfactory epithelium; pe, pancreatic epithelium; re, retina; Rp, Rathke's pouch; se, stomach epithelium; sm, skeletal muscle; tg, thyroid gland; tr, thymic rudiment. Scale bar of whole sagittal sections: 1mm. Scale bar of details: 250µm.

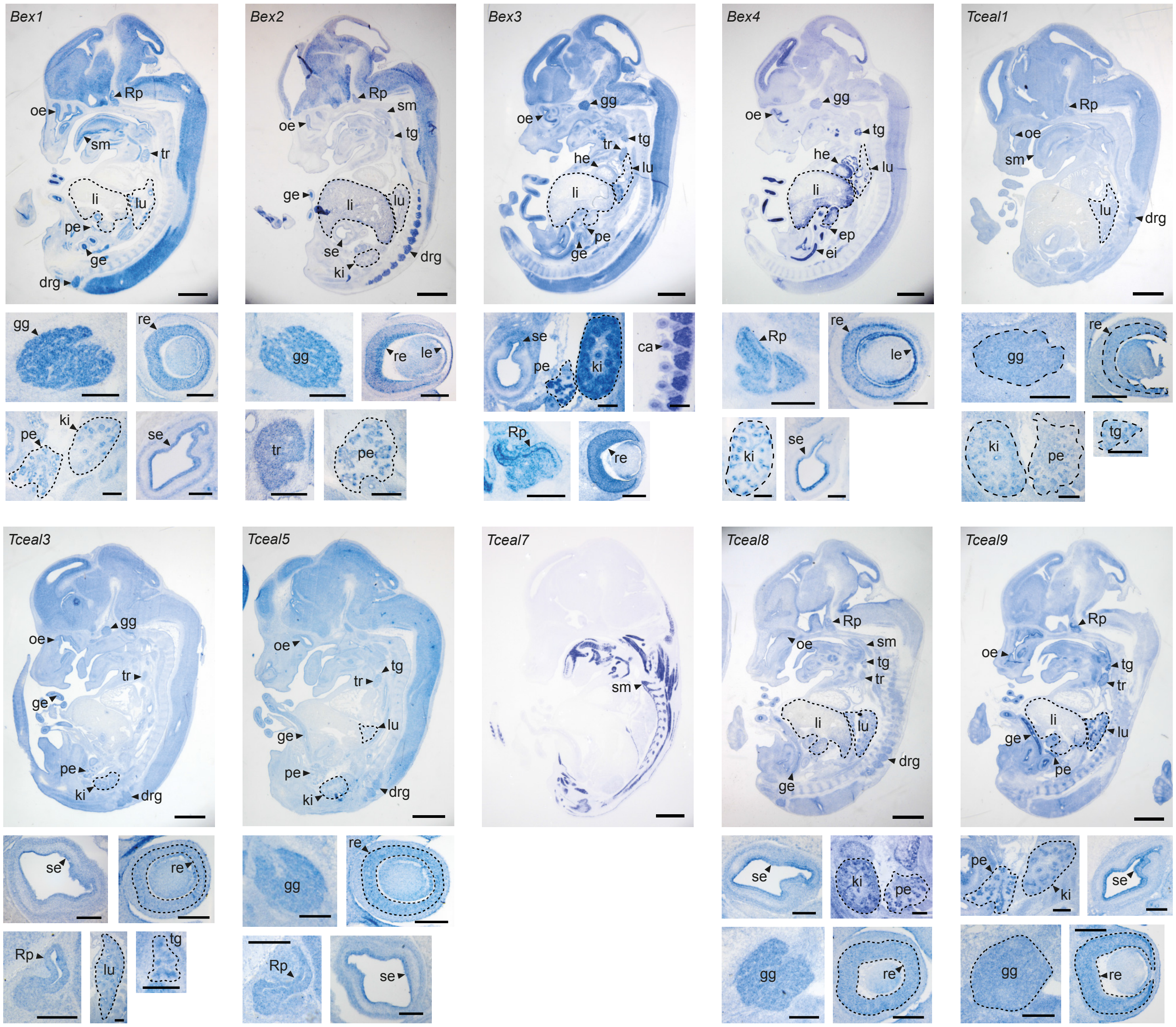

Fig. S8 *Bex3* and *Tceal7* genes, but not the ancestral HALEX element, induce cell proliferation in chicken neural tube. **A-B** Representative transverse sections of neural tubes from embryos electroporated at HH12 with pCIG, pCIGBex3, pCIGTceal7 and pCIGHALEX vectors and analysed at 24 hpe by immunostaining. GFP (green) and Sox2 (red) stain, respectively, the electroporated cells and the neural progenitors. **C-D** The percentage of GFP-positive electroporated cells positive for BrdU or Sox2 increases in pCIGBex3 and pCIGTceal7 electroporated embryos. Cell counting was carried out on 10-17 pictures obtained from 4 to 7 different chick embryos per experimental condition. Data represent the mean ± SEM; * $P < 0.05$, ** $P < 0.01$, unpaired Student's t tests.

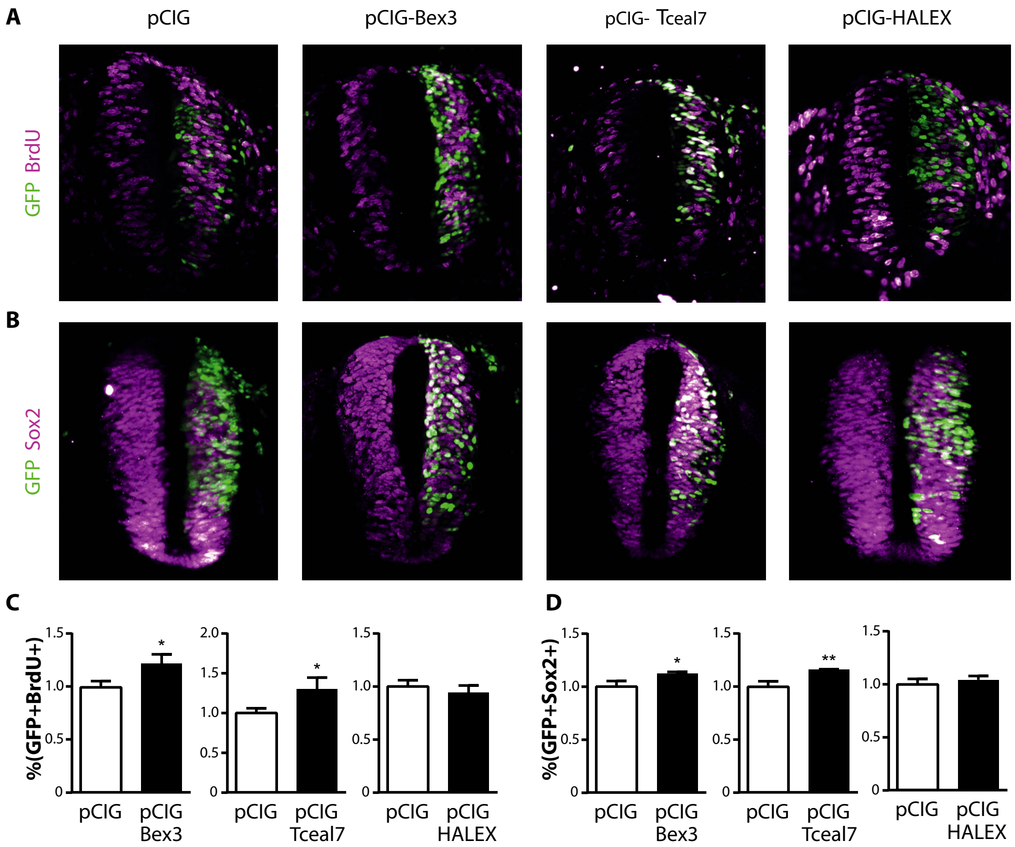

Fig. S9 The deletions introduced using CRISPR-Cas9 technology can be observed in the mRNA expressed from the *Bex3* mutant alleles. Brain expression of *Bex3* and *actin* from three adult male wild-type and mutant animals from both lines.

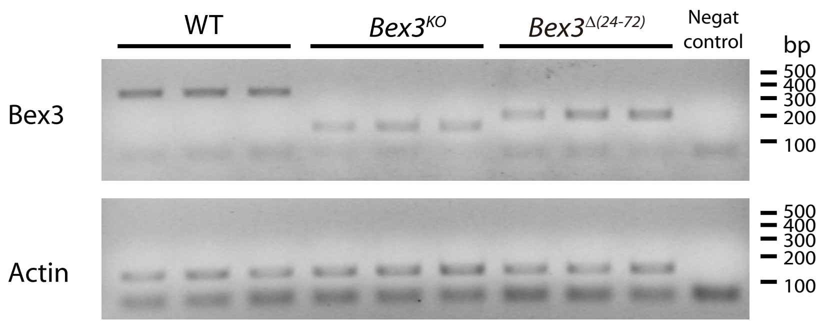

Fig. S10 CRISPR-Cas9-generated *Bex3* mutant alleles show subtle skull abnormalities. Morphometric analyses of skulls from wild-type and mutant *Bex3*^{KO} 6-week-old males **A** employing a total of 24 anatomical measurements **B** revealed that *Bex3* dysfunction led to cranial abnormalities in frontal bone and skull height **C**. Measurements were normalized to maximum skull length (D0) and expressed relative to controls (black horizontal line). Deviations of 5% with respect to controls are shown as dotted red and green horizontal lines. Results are presented as mean ± SEM ($N \geq 4$); * $P < 0.05$, one-way ANOVA.

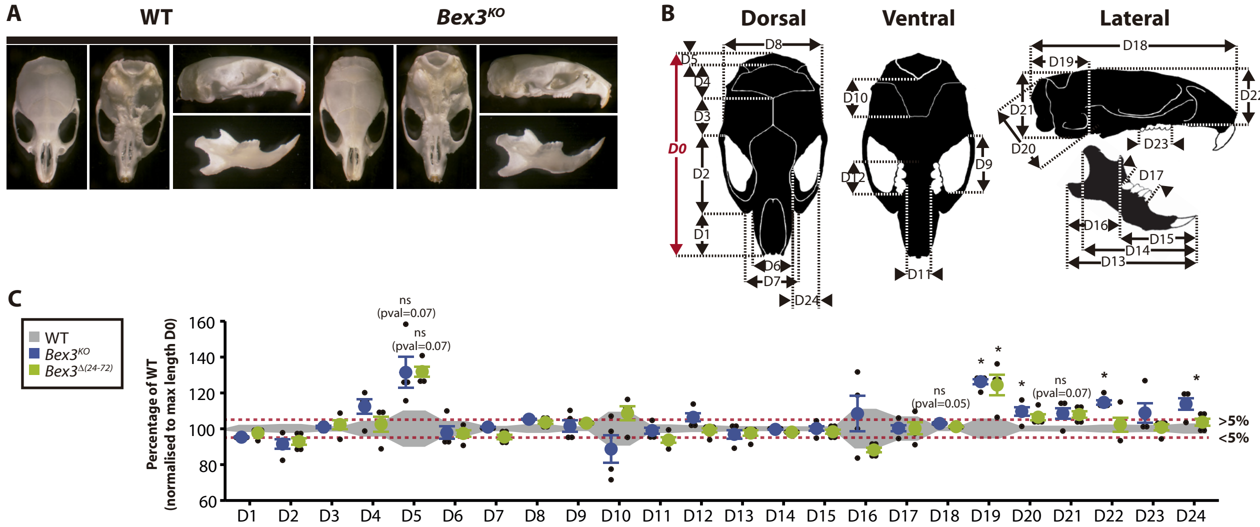

Fig. S11 *Bex3* mutant mice show normal acoustic startle reflex. To analyze the acoustic startle reflex, latency of response, latency of peak and peak amplitude of the startle response were evaluated. Results are presented as mean ± SEM ($N = 6$ per experimental group); one-way ANOVA.

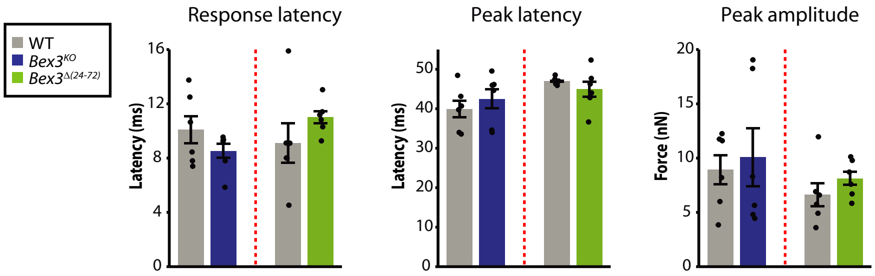

Fig. S12 *Bex3* deficiency leads to aberrant mTOR signalling in the brain. **A** Western blot analyses of whole brain lysates of adult $Bex3^{\Delta(24\text{-}72)}$ mice revealed abnormal phosphorylation ratios of some mTORC1 targets. Representative images. **B** Quantification relative to wild-type. Results are presented as mean ± SEM (*N* = 4 per experimental group); * $P < 0.05$, non-parametric Mann–Whitney test.

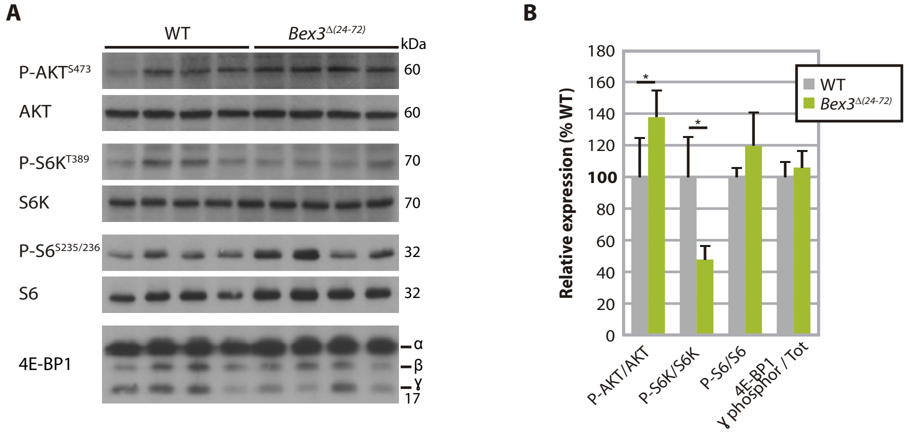

Table S1. Coding genes putatively derived from transposable elements in the human and mouse genomes.

Gene ID	% overlap	KnownGene	txCdsPredict score	Domesticated transposon	Reported	Conservation
Homo sapiens						
AK127846	100	uc002qao.3	2124	MER50-int (LTR retrotransposon)	-	Simiiformes
C5orf54 (ZBED8)	100	uc003lye.1	3097.83	Charlie11 (Cut and Paste DNA transposon)	[2]	
ERV3-1	100	uc011kdr.2	1829.5	HERV3-int (LTR retrotransposon)	[3]	
ERVFRD-1 (Syncitin-2)	57.05	uc003mzt.3	2188	MER50-int (LTR retrotransposon)	[4]	
ERVK6	99.93	uc032zcp.1	1583.5	HERVK-int (LTR retrotransposon)	[5]	
ERVMER34-1	53.81	uc003gzs.3	1965.67	MER34-int (LTR retrotransposon)	[6]	
ERVW-1 (Syncytin-1)	100	uc022ahe.2	1630.5	HERV17-int (LTR retrotransposon)	[7]	
FAM200A	94.02	uc003urb.3	2929.5	Charlie9 (Cut and Paste DNA transposon)	[8]	
FAM200B	100	uc003gof.4	2993	Charlie9 (Cut and Paste DNA transposon)	[8]	
GTF2IRD2	55.99	uc032zsc.1	4911	Charlie8 (Cut and Paste DNA transposon)	[9]	
GTF2IRD2B	100	uc003ubt.3	4988.33	Charlie8 (Cut and Paste DNA transposon)	[9]	
JRK	90.57	uc033cbj.1	3028.5	Tigger10 (Cut and Paste DNA transposon)	[10]	
L1TD1	53.81	uc001dae.5	5296.67	L1MEd, HAL1ME (LINE retrotransposon)	[11]	
MGC12965	54.66	uc001ove.3	1148	Tigger4 (Cut and Paste DNA transposon)	-	Primates
MOAP1 (PNMA4)	96.5	uc001ybj.3	2045.33	MamGyp-int (LTR retrotransposon)	[12]	
PNMA1	96.14	uc001xor.1	2279	MamGyp-int (LTR retrotransposon)	[12]	
PNMA2	92.7	uc003xez.2	2302	MamGyp-int (LTR retrotransposon)	[12]	
PNMA3	72.72	uc033far.1	2872.33	MamGyp-int (LTR retrotransposon)	[12]	
PNMA3	73.94	uc022cho.2	2854.33	MamGyp-int (LTR retrotransposon)	[12]	
PNMA5	60.76	uc010ntw.3	2491.67	MamGyp-int (LTR retrotransposon)	[12]	
PNMA6A	73.19	uc022chq.2	2306.67	MamGyp-int (LTR retrotransposon)	[12]	
PNMA6A	73.19	uc011myl.3	1972	MamGyp-int (LTR retrotransposon)	[12]	
SETMAR	71.84	uc011asp.2	3181.83	HSMAR1 (Cut and Paste DNA transposon)	[13]	
TCEAL7	76.32	uc004ekc.2	804	HAL1b (LINE retrotransposon)	-	Eutheria
TIGD1	100	uc002vsy.2	2366	Tigger1 (LTR retrotransposon)	[14]	
ZBED5	97.94	uc009ygh.3	3590.5	Charlie14a (LTR retrotransposon)	[2]	
ZMYM5	51.11	uc001umm.1	2329	Zaphod2 (Cut and Paste DNA transposon)	[15]	
ZMYM6 (ZBED7)	69.92	uc031txb.1	3128.17	Charlie10 (Cut and Paste DNA transposon)	[15]	
Mus musculus						
BC035947	94.78	uc011wnt.1	2345	RLTR6-int (LTR retrotransposon)	-	*Mus spretus*
Brip1os	79.18	uc007ksg.1	947	RLTR6-int (LTR retrotransposon)	-	*Mus* (genus)
Jrk	92.72	uc033gue.1	3457	Tigger10 (Cut and Paste DNA transposon)	[10]	
Moap1 (Pnma4)	96.13	uc011yqp.2	1593.5	MamGyp-int (LTR retrotransposon)	[12]	
Pnma1	96.14	uc007oel.1	2267	MamGyp-int (LTR retrotransposon)	[12]	
Pnma2	92.45	uc033grq.1	2386	MamGyp-int (LTR retrotransposon)	[12]	
Pnma3	71.97	uc009tla.1	2513	MamGyp-int (LTR retrotransposon)	[12]	
Skint5	67.71	uc009vcz.1	4936.5	MurERV4-int (LTR retrotransposon)	-	*Mus spretus*
Zbed5	78.51	uc008zto.1	2692.5	Charlie14a (LTR retrotransposon)	[2]	

Table S2. Altered expression of *BEX* and *TCEAL* genes in subjects with autism spectrum disorder or schizophrenia.

Gene symbol	Disorder	Fold change	*P* value	FDR	Probe	Tissue	Study (PMID) or GEO ID	Sample, cases vs. controls
BEX1	Autism	-1.60	3.59x10-4	**2.03x10-2**	ILMN_1702637*	cerebellum	GSE38322	14 ASD vs. 12 control
BEX1	Autism	N/A	N/A	N/A	N/A	dorsolateral prefrontal cortex	[16] (27685936)	34 ASD vs. 40 control
BEX1	Autism	-1.21	2.26x10-2	1.20x10-1	N/A	frontal and temporal cortex	[17] (27919067)	48 ASD vs. 49 control
BEX1	Autism	-1.43	N/A	**4.19x10-3**	N/A	prefrontal and anterior cingulate cortex (L4)	[18] (31097668)	15 ASD vs. 16 control
BEX1	Autism	-1.25	N/A	**4.92x10-2**	N/A	prefrontal and anterior cingulate cortex (L5/6)	[18] (31097668)	15 ASD vs. 16 control
BEX1	Autism	-1.74	N/A	**1.47x10-3**	N/A	prefrontal and anterior cingulate cortex (Neu-NRGN-II)	[18] (31097668)	15 ASD vs. 16 control
BEX1	Autism	-1.21	N/A	**2.28x10-2**	N/A	prefrontal and anterior cingulate cortex (IN-PV)	[18] (31097668)	15 ASD vs. 16 control
BEX1	Autism	-1.31	N/A	**5.90x10-3**	N/A	prefrontal and anterior cingulate cortex (IN-SST)	[18] (31097668)	15 ASD vs. 16 control
BEX1	Autism	-1.29	N/A	**1.03x10-4**	N/A	prefrontal and anterior cingulate cortex (IN-VIP)	[18] (31097668)	15 ASD vs. 16 control
BEX1	Autism	-1.24	N/A	**6.95x10-3**	N/A	prefrontal and anterior cingulate cortex (IN-SV2C)	[18] (31097668)	15 ASD vs. 16 control
BEX1	Schizophrenia	-1.08	8.22x10-4	**5.90x10-2**	8174201	cerebellum	GSE35978	44 scz vs. 50 control
BEX1	Schizophrenia	-1.33	1.42x10-4	**1.18x10-2**	218332_at	hippocampus	GSE53987	15 scz vs. 18 control
BEX1	Schizophrenia	-1.06	3.38x10-2	3.34x10-1	218332_at	prefrontal cortex	GSE53987	15 scz vs. 19 control
BEX1	Schizophrenia	-1.14	2.84x10-2	2.92x10-1	218332_at	striatum	GSE53987	18 scz vs. 18 control
BEX1	Schizophrenia	-1.07	8.57x10-4	**5.92x10-2**	11719475_a_at	dorsolateral prefrontal cortex	GSE87610	65 scz vs. 72 control
BEX1	Schizophrenia	-1.09	N/A	**4.65x10-3**	218332_at	prefrontal cortex	[19] (26818902)	122 scz vs. 124 control
BEX1	Schizophrenia	N/A	4.32x10-4	**9.26x10-2**	N/A	anterior cingulate cortex	[20] (25113377)	31 scz vs. 26 control
BEX2	Autism	-1.53	5.21x10-4	**2.31x10-2**	ILMN_2181892	cerebellum	GSE38322	14 ASD vs. 12 control
BEX2	Autism	-1.20	4.12x10-2	1.67x10-1	N/A	frontal and temporal cortex	[17] (27919067)	48 ASD vs. 49 control
BEX2	Schizophrenia	-1.26	1.21x10-3	**2.59x10-2**	224367_at	hippocampus	GSE53987	15 scz vs. 18 control
BEX2	Schizophrenia	-1.09	1.15x10-2	1.97x10-1	11718848_a_at	dorsolateral prefrontal cortex	GSE87610	65 scz vs. 72 control
BEX3	Autism	-1.48	1.43x10-2	1.25x10-1	ILMN_1729208*	cerebellum	GSE38322	14 ASD vs. 12 control
BEX3	Autism	-1.18	3.66x10-3	**5.15x10-2**	N/A	frontal and temporal cortex	[17] (27919067)	48 ASD vs. 49 control
BEX3	Autism	-1.05	2.35x10-2	1.61x10-1	N/A	frontal and temporal cortex	[21] (30545856)	51 ASD vs. 936 control
BEX3	Autism	-1.25	N/A	**4.50x10-2**	N/A	prefrontal and anterior cingulate cortex (L4)	[18] (31097668)	15 ASD vs. 16 control
BEX3	Autism	-1.62	N/A	**4.12x10-2**	N/A	prefrontal and anterior cingulate cortex (Neu-NRGN-II)	[18] (31097668)	15 ASD vs. 16 control
BEX3	Autism	-1.29	N/A	**5.45x10-4**	N/A	prefrontal and anterior cingulate cortex (IN-VIP)	[18] (31097668)	15 ASD vs. 16 control
BEX3	Autism	-1.34	N/A	**3.81x10-3**	N/A	prefrontal and anterior cingulate cortex (IN-SV2C)	[18] (31097668)	15 ASD vs. 16 control
BEX3	Schizophrenia	-1.03	4.77x10-2	3.39x10-1	8169028	cerebellum	GSE35978	44 scz vs. 50 control
BEX3	Schizophrenia	-1.28	1.88x10-4	**1.28x10-2**	217963_s_at	hippocampus	GSE53987	15 scz vs. 18 control
BEX3	Schizophrenia	-1.06	4.56x10-2	3.76x10-1	217963_s_at	prefrontal cortex	GSE53987	15 scz vs. 19 control
BEX3	Schizophrenia	-1.11	1.81x10-2	2.67x10-1	217963_s_at	striatum	GSE53987	18 scz vs. 18 control
BEX3	Schizophrenia	-1.28	5.00x10-3	N/A	217963_s_at	dorsolateral prefrontal cortex	[22] (24886351)	8 scz vs. 7 control
BEX3	Schizophrenia	-1.06	N/A	**5.41x10-3**	217963_s_at	prefrontal cortex	[19] (26818902)	122 scz vs. 124 control
BEX4	Autism	-1.65	6.17x10-3	**7.82x10-2**	ILMN_2351638	cerebellum	GSE38322	14 ASD vs. 12 control

Gene symbol	Disorder	Fold change	*P* value	FDR	Probe	Tissue	Study (PMID) or GEO ID	Sample, cases vs. controls
BEX4	Autism	1.10	4.26x10-2	6.72x10-1	ILMN_1773504	occipital cortex	GSE38322	6 ASD vs. 4 control
BEX4	Autism	-1.14	3.45x10-2	4.68x10-1	N/A	cerebellum	[17] (27919067)	48 ASD vs. 49 control
BEX4	Autism	-1.11	2.43x10-2	1.25x10-1	N/A	frontal and temporal cortex	[17] (27919067)	48 ASD vs. 49 control
BEX4	Autism	-1.11	3.00x10-2	N/A	ILMN_1804798	prefrontal and temporal cortex	[23] (29859039)	15 ASD vs. 16 control
BEX4	Schizophrenia	-1.30	4.70x10-4	**1.75x10-2**	215440_s_at	hippocampus	GSE53987	15 scz vs. 18 control
BEX4	Schizophrenia	-1.16	2.46x10-2	2.83x10-1	215440_s_at	striatum	GSE53987	18 scz vs. 18 control
BEX5	Autism	-1.93	3.78x10-4	**2.80x10-2**	ILMN_1806473	cerebellum and occipital cortex	[24] (22984548)	9 ASD vs. 9 control
BEX5	Autism	-2.07	8.63x10-5	**1.32x10-2**	ILMN_1806473	cerebellum	GSE38322	14 ASD vs. 12 control
BEX5	Autism	-2.30	1.36x10-2	5.78x10-1	ILMN_1806473	occipital cortex	GSE38322	6 ASD vs. 4 control
BEX5	Autism	-1.27	2.09x10-2	4.44x10-1	N/A	cerebellum	[17] (27919067)	48 ASD vs. 49 control
BEX5	Autism	-1.29	3.35x10-3	**4.92x10-2**	N/A	frontal and temporal cortex	[17] (27919067)	48 ASD vs. 49 control
BEX5	Autism	-1.49	3.53x10-2	N/A	ILMN_1806473	prefrontal and temporal cortex	[23] (29859039)	15 ASD vs. 16 control
BEX5	Autism	-1.10	2.70x10-2	1.74x10-1	N/A	frontal and temporal cortex	[21] (30545856)	51 ASD vs. 936 control
BEX5	Schizophrenia	-1.13	1.23x10-4	**2.58x10-2**	8174141	cerebellum	GSE35978	44 scz vs. 50 control
BEX5	Schizophrenia	-1.40	7.24x10-4	**2.05x10-2**	229963_at	hippocampus	GSE53987	15 scz vs. 18 control
BEX5	Schizophrenia	-1.10	5.00x10-2	3.86x10-1	229963_at	prefrontal cortex	GSE53987	15 scz vs. 19 control
BEX5	Schizophrenia	-1.24	1.10x10-2	2.50x10-1	229963_at	striatum	GSE53987	18 scz vs. 18 control
BEX5	Schizophrenia	-1.15	8.11x10-4	**5.71x10-2**	11725493_s_at	dorsolateral prefrontal cortex	GSE87610	65 scz vs. 72 control
TCEAL1	Autism	-1.62	1.86x10-4	**1.65x10-2**	ILMN_2398403*	cerebellum	GSE38322	14 ASD vs. 12 control
TCEAL1	Autism	-1.65	3.94x10-2	6.62x10-1	ILMN_2398408*	occipital cortex	GSE38322	6 ASD vs. 4 control
TCEAL1	Autism	-1.16	1.55x10-2	4.37x10-1	N/A	cerebellum	[17] (27919067)	48 ASD vs. 49 control
TCEAL1	Autism	-1.10	2.91x10-2	1.38x10-1	N/A	frontal and temporal cortex	[17] (27919067)	48 ASD vs. 49 control
TCEAL1	Schizophrenia	-1.06	2.90x10-3	1.06x10-1	8169049	cerebellum	GSE35978	44 scz vs. 50 control
TCEAL1	Schizophrenia	-1.17	1.08x10-2	**8.21x10-2**	204045_at	hippocampus	GSE53987	15 scz vs. 18 control
TCEAL1	Schizophrenia	-1.18	4.48x10-3	2.30x10-1	204045_at	striatum	GSE53987	18 scz vs. 18 control
TCEAL2	Autism	-1.12	N/A	**1.52x10-3**	N/A	prefrontal and anterior cingulate cortex (L4)	[18] (31097668)	15 ASD vs. 16 control
TCEAL2	Schizophrenia	-1.13	4.10x10-2	1.77x10-1	211276_at	hippocampus	GSE53987	15 scz vs. 18 control
TCEAL2	Schizophrenia	-1.07	2.60x10-2	2.75x10-1	11727253_at	dorsolateral prefrontal cortex	GSE87610	65 scz vs. 72 control
TCEAL2	Schizophrenia	1.03	2.05x10-3	**1.61x10-2**	N/A	frontal and temporal cortex	[21] (30545856)	559 scz vs. 936 control
TCEAL3	Autism	N/A	N/A	N/A	N/A	dorsolateral prefrontal cortex	[16] (27685936)	34 ASD vs. 40 control
TCEAL3	Schizophrenia	-1.15	2.34x10-3	**3.59x10-2**	227279_at	hippocampus	GSE53987	15 scz vs. 18 control
TCEAL3	Schizophrenia	-1.25	4.52x10-5	**1.16x10-2**	11725131_x_at*	dorsolateral prefrontal cortex	GSE87610	65 scz vs. 72 control
TCEAL4	Autism	-1.10	4.02x10-2	N/A	ILMN_1748625	prefrontal and temporal cortex	[23] (29859039)	15 ASD vs. 16 control
TCEAL4	Schizophrenia	-1.17	8.89x10-4	**2.23x10-2**	202371_at	hippocampus	GSE53987	15 scz vs. 18 control
TCEAL4	Schizophrenia	-1.07	1.76x10-2	2.65x10-1	202371_at	striatum	GSE53987	18 scz vs. 18 control
TCEAL4	Schizophrenia	-1.13	4.27x10-3	1.28x10-1	11745071_a_at	dorsolateral prefrontal cortex	GSE87610	65 scz vs. 72 control
TCEAL5	Autism	-1.17	3.10x10-2	1.91x10-1	ILMN_1749073	cerebellum	GSE38322	14 ASD vs. 12 control
TCEAL6	Autism	-1.48	1.82x10-2	1.42x10-1	ILMN_1729165*	cerebellum	GSE38322	14 ASD vs. 12 control
TCEAL6	Schizophrenia	-1.47	6.37x10-6	**4.25x10-3**	11737036_at*	dorsolateral prefrontal cortex	GSE87610	65 scz vs. 72 control
TCEAL6	Schizophrenia	1.03	1.68x10-2	**7.69x10-2**	N/A	frontal and temporal cortex	[21] (30545856)	559 scz vs. 936 control
TCEAL7	Autism	-1.71	1.30x10-3	**3.41x10-2**	ILMN_1753525	cerebellum	GSE38322	14 ASD vs. 12 control
TCEAL7	Autism	-1.24	1.69x10-2	5.86x10-1	ILMN_2084043	occipital cortex	GSE38322	6 ASD vs. 4 control
TCEAL7	Autism	-1.18	3.06x10-3	**4.76x10-2**	N/A	frontal and temporal cortex	[17] (27919067)	48 ASD vs. 49 control
TCEAL7	Autism	-1.06	4.42x10-2	2.29x10-1	N/A	frontal and temporal cortex	[21] (30545856)	51 ASD vs. 936 control
TCEAL7	Schizophrenia	-1.09	4.20x10-3	1.23x10-1	8169015	cerebellum	GSE35978	44 scz vs. 50 control
TCEAL7	Schizophrenia	-1.35	1.23x10-4	**1.12x10-2**	227705_at	hippocampus	GSE53987	15 scz vs. 18 control
TCEAL7	Schizophrenia	-1.14	2.46x10-2	2.83x10-1	227705_at	striatum	GSE53987	18 scz vs. 18 control

Gene symbol	Disorder	Fold change	*P* value	FDR	Probe	Tissue	Study (PMID) or GEO ID	Sample, cases vs. controls
TCEAL7	Schizophrenia	-1.04	2.36x10-5	**5.21x10-4**	N/A	frontal and temporal cortex	[21] (30545856)	559 scz vs. 936 control
TCEAL8	Autism	-1.36	1.05x10-3	**3.04x10-2**	ILMN_2402272*	cerebellum	GSE38322	14 ASD vs. 12 control
TCEAL8	Autism	-1.56	5.82x10-3	5.53x10-1	ILMN_2402272*	occipital cortex	GSE38322	6 ASD vs. 4 control
TCEAL8	Autism	-1.13	2.36x10-2	4.49x10-1	N/A	cerebellum	[17] (27919067)	48 ASD vs. 49 control
TCEAL8	Autism	-1.11	2.74x10-2	N/A	ILMN_2402272	prefrontal and temporal cortex	[23] (29859039)	15 ASD vs. 16 control
TCEAL8	Schizophrenia	-1.24	1.48x10-3	**2.86x10-2**	224819_at	hippocampus	GSE53987	15 scz vs. 18 control
TCEAL8	Schizophrenia	-1.18	1.69x10-2	2.63x10-1	224819_at	striatum	GSE53987	18 scz vs. 18 control
TCEAL9	Schizophrenia	1.15	3.12x10-3	1.59x10-1	217975_at	prefrontal cortex	GSE53987	15 scz vs. 19 control

N/A, data not available; FDR, False Discovery Rate; scz, schizophrenia cases; ASD, autism spectrum disorder cases; L4, layer 4 excitatory neurons; L5/6, layer 5/6 corticofugal projection neurons; Neu-NRGN, neurogranin–expressing neurons; IN-PV, parvalbumin interneurons; IN-SST, somatostatin interneurons;IN-VIP, vasoactive intestinal polypeptide–expressing interneurons; IN-SV2C, synaptic vesicle glycoprotein 2C-expressing interneurons. Experiments overcoming multiple testing corrections at 10% FDR are highlighted in bold. *Genes showing significant differential expression in several independent probe sets, data shown corresponding to the probe with the highest FC.

Table S3. Enrichment of differential gene expression in *BEX* and *TCEAL* gene families in subjects with autism spectrum disorder or schizophrenia.

Study (PMID) or GEO ID	Disorder	Tissue	# genes analyzed	# DE genes	*BEX* genes	*TCEAL* genes	TOTAL	*BEX* enrichment (*P* value)	*TCEAL* enrichment (*P* value)	*BEX+TCEAL* enrichment (*P* value)
[21] (30545856)	Autism	frontal and temporal cortex	25772	5250	2	1	1	**1.10x10-2**	1	**3.04x10-2**
[24] (22984548)	Autism	cerebellum and occipital cortex	18626	41	1	0	7	**2.17x10-3**	7.92x10-1	8.39x10-2
GSE38322	Autism	cerebellum	20762	6312	5	5	4	8.34x10-2	2.30x10-1	**4.60x10-2**
GSE38322	Autism	occipital cortex	20762	2341	2	3	4	2.19x10-1	4.96x10-1	2.30x10-1
[16] (27685936)	Autism	prefrontal cortex	12557	1775	1	1	10	**2.59x10-3**	1.04x10-1	**1.86x10-3**
[17] (27919067)	Autism	cerebellum	13190	1337	2	2	5	1.01x10-1	7.14x10-2	**1.52x10-2**
[17] (27919067)	Autism	frontal and temporal cortex	13543	3973	5	2	2	5.33x10-1	7.46x10-1	6.09x10-1
[23] (29859039)	Autism	frontal and temporal cortex	12632	2254	2	2	3	**3.99x10-2**	4.82x10-1	8.06x10-2
[18] (31097668)	Autism	prefrontal and anterior cingulate cortex (L4)	1391	94	2	1	3	**3.95x10-2**	4.68x10-1	6.31x10-2
[18] (31097668)	Autism	prefrontal and anterior cingulate cortex (L5/6)	1391	8	1	0	1	**2.85x10-2**	1	7.79x10-2
[18] (31097668)	Autism	prefrontal and anterior cingulate cortex (Neu-NRGN-II)	1391	25	2	0	2	**3.00x10-3**	1	**2.47x10-2**
[18] (31097668)	Autism	prefrontal and anterior cingulate cortex (IN-PV)	1391	27	1	0	1	9.35x10-2	1	2.41x10-1
[18] (31097668)	Autism	prefrontal and anterior cingulate cortex (IN-SST)	1391	27	1	0	1	9.35x10-2	1	2.41x10-1
[18] (31097668)	Autism	prefrontal and anterior cingulate cortex (IN-VIP)	1391	78	2	0	2	**2.78x10-2**	1	1.83x10-1
[18] (31097668)	Autism	prefrontal and anterior cingulate cortex (IN-SV2C)	1391	32	2	0	2	**4.91x10-3**	1	**3.93x10-2**
[21] (30545856)	Schizophrenia	frontal and temporal cortex	25772	7840	0	3	5	**9.70x10-5**	**8.32x10-4**	**3.36x10-7**
GSE35978	Schizophrenia	cerebellum	20359	3545	3	2	11	**1.43x10-2**	1.55x10-1	**8.84x10-3**
GSE53987	Schizophrenia	hippocampus	22187	3497	5	6	8	8.27x10-2	9.04x10-1	4.04x10-1
GSE53987	Schizophrenia	prefrontal cortex	22187	5083	3	1	4	5.06x10-1	1	8.61x10-1
GSE53987	Schizophrenia	striatum	22187	5416	4	4	1	**9.00x10-3**	1	6.82x10-2
GSE87610	Schizophrenia	dorsolateral prefrontal cortex	20034	5258	3	4	2	**4.74x10-2**	1	1.27x10-1
[22] (24886351)	Schizophrenia	dorsolateral prefrontal cortex	20955	2757 probes	1	0	1	**2.22x10-2**	**2.71x10-2**	**1.48x10-3**
[19] (26818902)	Schizophrenia	prefrontal cortex	22277 probes	690	2	0	7	2.70x10-1	8.71x10-1	5.66x10-1
[20] (25113377)	Schizophrenia	anterior cingulate cortex	14454	105	1	0	3	1	5.48x10-1	8.47x10-1

DE genes: differentially expressed genes. Enrichment calculated using an hypergeometric test for the 5 BEX genes and the 9 TCEAL genes. Significant *P* values are highlighted in bold.

Table S4. Primers and reconstructed gene sequences used in this work.

Experiment	Name	Sequence
Generation of CRISPR-Cas9 sgRNA guides targeting *Bex3*	sgRNA1-F	agggGGACAGGAAGACCGCCCTGT
	sgRNA1-R	aaacACAGGGCGGTCTTCCTGTCC
	sgRNA2-F	agggCAGATGAATGACGGGTT
	sgRNA2-R	aaacAACCCGTCATTCATCTG
Template preparation for IDA // Genotyping of *Bex3* mutant lines	Bex3F-Ext	CCTGTCTAGGACCCCTGTGA
	Bex3R-Ext	GCGGGAGTCACAGTATGGAT
	Bex3F-Int	AGCCCCCACTCCACACTACT
	Bex3R-Int	TGGTGATCGTGGTGGTTAGA
RT-PCR *Bex3*	Bex3_RT_F	CCAATGTCCACCAGGAAAAC
	Bex3_RT_R	AGGCATAAGGCAGAATTCATCA
RT-PCR *actin*	act_RT_F	GGCTGTATTCCCCTCCATCG
	act_RT_R	CCAGTTGGTAACAATGCCATG
Riboprobe for *in situ* hyridization of *Bex1*	Bex1F	CATCATGACCACCATGATGAG
	Bex1R	GGTTCACAATAGGTAATACGG
Riboprobe for *in situ* hyridization of *Bex2*	Bex2F	CATCATGACCACCATGATGAG
	Bex2R	GGTTCACAATATATACTGAGC
Riboprobe for *in situ* hyridization of *Bex3*	Bex3F	CGAAGAGATGGAGCAGCCCC
	Bex3R	CATGCTAATGGGCAACACTG
Riboprobe for *in situ* hyridization of *Bex4*	Bex4F	GGCAAGGATAGGCCCAGGAG
	Bex4R	ATGATTGTCAGGTTCCGGGG
Riboprobe for *in situ* hyridization of *Tceal1*	Tceal1F	CAGCCTGCAGTGGAGCAGTC
	Tceal1R	TAAGGGCGGCTCCGTTTTGC
Riboprobe for *in situ* hyridization of *Tceal3*	Tceal3F	GTTCACGTGACCGGCCAGGC
	Tceal3R	GACAGGCCTTTGCCTTCTCC
Riboprobe for *in situ* hyridization of *Tceal5*	Tceal5F	GAAGTCTCTCTCTTCCAGGT
	Tceal5R	CCATGGTGCAATTAGTCTTTG
Riboprobe for *in situ* hyridization of *Tceal7*	Tceal7F	AGCCCAAGGGCAGTGAGGCA
	Tceal7R	CAGGTGACAGTTGACGTGCC
Riboprobe for *in situ* hyridization of *Tceal8*	Tceal8F	CGAGAACGAAGGAACACCCC
	Tceal8R	AAGCCAGAGGGAATTCCAGG
Riboprobe for *in situ* hyridization of *Tceal9*	Tceal9F	GGAGGATGAGCCAAAGCCTG
	Tceal9F	CATACAAAGGTACTCCTGTC
HALEX ancestral gene reconstruction	HALEX	ATGCAGACCTATTATTCAAAAGAGGAGCGGGGGAAAGAAGAAAGACGGCACTCAATGC<br>AGAAGACCGACGAGGAACCAAGCCTGGAAGAGAATATCACCCAGGGCACAGAGGAAA<br>ACAGCTCCCAGGTGCTGCACGCCATCGAAAAGCTGAACAAAAACATGAACTCTATCAAG<br>CAGGAGCTGAAAGACGAAATGATTAAGCAGCAGAATGAGATGAAAAGAGAGATCGCTG<br>AACTGAGGAAGCAGATTGAAAACCAGAACAACATCCTGCACTCCAATCACCGACATTCC<br>CGAGACCTGCTGTACCTG

Supplementary references

1. Tarver JE, Dos Reis M, Mirarab S, Moran RJ, Parker S, O'Reilly JE, et al. The interrelationships of placental mammals and the limits of phylogenetic inference. Genome Biol Evol. 2016;8:330–44.
2. Aravind L. The BED finger, a novel DNA-binding domain in chromatin-boundary-element-binding proteins and transposases. Trends Biochem Sci. 2000;25:421–3.
3. Boyd MT, Bax CMR, Bax BE, Bloxam DL, Weiss RA. The Human Endogenous Retrovirus ERV-3 Is Upregulated in Differentiating Placental Trophoblast Cells. Virology. 1993;196:905–9.
4. Blaise S, de Parseval N, Bénit L, Heidmann T. Genomewide screening for fusogenic human endogenous retrovirus envelopes identifies syncytin 2, a gene conserved on primate evolution. Proc Natl Acad Sci U S A. 2003;100:13013–8.
5. Mayer J, Sauter M, Racz A, Scherer D, Mueller-Lantzsch N, Meese E. An almost-intact human endogenous retrovirus K on human chromosome 7. Nat Genet. 1999;21:257–8.
6. Heidmann O, Béguin A, Paternina J, Berthier R, Deloger M, Bawa O, et al. HEMO, an ancestral endogenous retroviral envelope protein shed in the blood of pregnant women and expressed in pluripotent stem cells and tumors. Proc Natl Acad Sci U S A. 2017;114:E6642–51.
7. Mi S, Lee X, Li X, Veldman GM, Finnerty H, Racie L, et al. McCoy, Syncytin is a captive retroviral envelope protein involved in human placental morphogenesis. Nature. 2000;403:785–9.
8. Smit AF. Interspersed repeats and other mementos of transposable elements in mammalian genomes. Curr Opin Genet Dev. 1999;9:657–63.
9. Tipney HJ, Hinsley TA, Brass A, Metcalfe K, Donai D, Tassabehji M. Isolation and characterisation of GTF21RD2, a novel fusion gene and member of the TFII-I family of transcription factors, deleted in Williams-Beuren syndrome. Eur J Hum Genet. 2004;12:551–60.
10. Toth M, Grimsby J, Buzsaki G, Donovan GP. Epileptic seizures caused by inactivation of a novel gene, jerky, related to centromere binding protein-B in transgenic mice. Nat Genet. 1995;11:71–5.

11. McLaughlin RN, Young JM, Yang L, Neme R, Wichman HA, Malik HS. Positive selection and multiple losses of the LINE-1-derived L1TD1 gene in mammals suggest a dual role in genome defense and pluripotency. PLoS Genet. 2014;10:e1004531.
12. Campillos M, Doerks T, Shah PK, Bork P. Computational characterization of multiple Gag-like human proteins. Trends Genet. 2006;22:585–9.
13. Cordaux R, Udit S, Batzer MA, Feschotte C. Birth of a chimeric primate gene by capture of the transposase gene from a mobile element. Proc Natl Acad Sci U S A. 2006;103:8101–6.
14. Smit AFA, Riggs AD. Tiggers and other DNA transposon fossils in the human genome. Proc Natl Acad Sci U S A. 1996;93:1443–8.
15. Kojima KK, Jurka J. Crypton transposons: Identification of new diverse families and ancient domestication events. Mob DNA. 2011;2:12.
16. Liu X, Han D, Somel M, Jiang X, Hu H, Guijarro P, et al. Disruption of an Evolutionarily Novel Synaptic Expression Pattern in Autism. PLoS Biol. 2016;14:e1002558.
17. Parikshak NN, Swarup V, Belgard TG, Irimia M, Ramaswami G, Gandal MJ, et al. Genome-wide changes in lncRNA, splicing, and regional gene expression patterns in autism. Nature. 2016;540:423–7.
18. Velmeshev D, Schirmer L, Jung D, Haeussler M, Perez Y, Mayer S, et al. Single-cell genomics identifies cell type–specific molecular changes in autism. Science. 2019;364:685–9.
19. Qin W, Liu C, Sodhi M, Lu H. Meta-analysis of sex differences in gene expression in schizophrenia. BMC Syst Biol. 2016;10:9.
20. Zhao Z, Xu J, Chen J, Kim S, Reimers M, Bacanu S-A, et al. Transcriptome sequencing and genome-wide association analyses reveal lysosomal function and actin cytoskeleton remodeling in schizophrenia and bipolar disorder. Mol Psychiatry. 2015;20:563–72.

21. Gandal MJ, Zhang P, Hadjimichael E, Walker RL, Chen C, Liu S, et al. Transcriptome-wide isoform-level dysregulation in ASD, schizophrenia, and bipolar disorder. Science. 2018;362:pii:eaat8127.
22. Hagihara H, Ohira K, Takao K, Miyakawa T. Transcriptomic evidence for immaturity of the prefrontal cortex in patients with schizophrenia. Mol Brain. 2014;7:41.
23. Schwede M, Nagpal S, Gandal MJ, Parikshak NN, Mirnics K, Geschwind DH, et al. Strong correlation of downregulated genes related to synaptic transmission and mitochondria in post-mortem autism cerebral cortex. J Neurodev Disord. 2018;10:18.
24. Ginsberg MR, Rubin RA, Falcone T, Ting AH, Natowicz MR. Brain transcriptional and epigenetic associations with autism. PLoS One. 2012;7:e44736.
